# (*Z*)-1-Acetyl-3-[2-oxo-1-phenyl-2-(3-pyrid­yl)ethyl­idene]indolin-2-one

**DOI:** 10.1107/S1600536810030874

**Published:** 2010-08-11

**Authors:** Hoong-Kun Fun, Jia Hao Goh, Haitao Yu, Yan Zhang

**Affiliations:** a X-ray Crystallography Unit, School of Physics, Universiti Sains Malaysia, 11800 USM, Penang, Malaysia; b School of Chemistry and Chemical Engineering, Nanjing University, Nanjing 210093, People’s Republic of China

## Abstract

The title compound, C_23_H_16_N_2_O_3_, exists in a *Z* configuration with respect to the acyclic C=C bond. The pyridine and phenyl rings are oriented at dihedral angles of 72.97 (4) and 45.05 (4)°, respectively, with respect to the almost planar indoline ring system [maximum deviation 0.080 (1) Å]. The pyridine and phenyl rings are oriented almost perpendicular to each other [dihedral angle 88.93 (5)°]. In the crystal, mol­ecules are inter­connected into a three-dimensional framework *via* inter­molecular C—H⋯O and C—H⋯N hydrogen bonds and weak π–π inter­actions [centroid–centroid distance = 3.681 (1) Å].

## Related literature

For general background and applications of indoline compounds, see: Aanandhi *et al.* (2008 ▶); Lawrence *et al.* (2008 ▶); Muthukumar *et al.* (2008 ▶); Wang *et al.* (2005 ▶); Xue *et al.* (2000 ▶); Yu *et al.* (2010 ▶); Zhang & Panek (2009 ▶); Zhang *et al.* (2004**a* ▶,b* ▶). For related indoline structures, see: Fun *et al.* (2010**a* ▶,b* ▶); Usman *et al.* (2001 ▶,2002 ▶). For the stability of the temperature controller used in the data collection, see: Cosier & Glazer (1986 ▶).
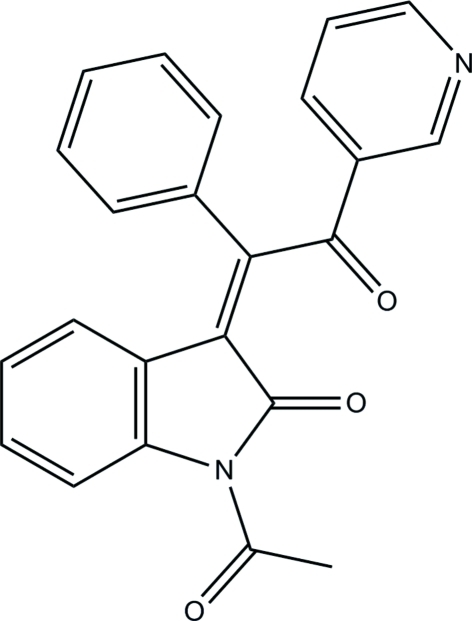


## Experimental

### 

#### Crystal data

C_23_H_16_N_2_O_3_
*M* *_r_* = 368.38
Triclinic, 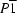

*a* = 7.9259 (19) Å
*b* = 9.086 (2) Å
*c* = 12.431 (3) Å
α = 84.804 (7)°
β = 87.064 (7)°
γ = 76.843 (6)°
*V* = 867.7 (4) Å^3^
*Z* = 2
Mo *K*α radiation
μ = 0.10 mm^−1^
*T* = 100 K
0.23 × 0.19 × 0.16 mm

#### Data collection

Bruker APEXII DUO CCD area-detector diffractometer
Absorption correction: multi-scan (*SADABS*; Bruker, 2009 ▶) *T*_min_ = 0.978, *T*_max_ = 0.985
19600 measured reflections
5017 independent reflections
4237 reflections with *I* > 2σ(*I*)
*R*_int_ = 0.028

#### Refinement

*R*[*F* ^2^ > 2σ(*F* ^2^)] = 0.040
*wR*(*F* ^2^) = 0.116
*S* = 1.04
5017 reflections
254 parameters
H-atom parameters constrained
Δρ_max_ = 0.44 e Å^−3^
Δρ_min_ = −0.20 e Å^−3^

### 

Data collection: *APEX2* (Bruker, 2009 ▶); cell refinement: *SAINT* (Bruker, 2009 ▶); data reduction: *SAINT*; program(s) used to solve structure: *SHELXTL* (Sheldrick, 2008 ▶); program(s) used to refine structure: *SHELXTL*; molecular graphics: *SHELXTL*; software used to prepare material for publication: *SHELXTL* and *PLATON* (Spek, 2009 ▶).

## Supplementary Material

Crystal structure: contains datablocks global, I. DOI: 10.1107/S1600536810030874/ci5150sup1.cif

Structure factors: contains datablocks I. DOI: 10.1107/S1600536810030874/ci5150Isup2.hkl

Additional supplementary materials:  crystallographic information; 3D view; checkCIF report

## Figures and Tables

**Table 1:** Hydrogen-bond geometry (Å, °)

*D*—H⋯*A*	*D*—H	H⋯*A*	*D*⋯*A*	*D*—H⋯*A*
C12—H12*A*⋯O3^i^	0.93	2.37	3.2518 (16)	158
C14—H14*A*⋯O2^ii^	0.93	2.53	3.2381 (16)	133
C20—H20*A*⋯N2^iii^	0.93	2.58	3.3238 (17)	137

Symmetry codes: (i) 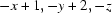; (ii) 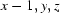; (iii) 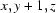.

**Fig. 1.**
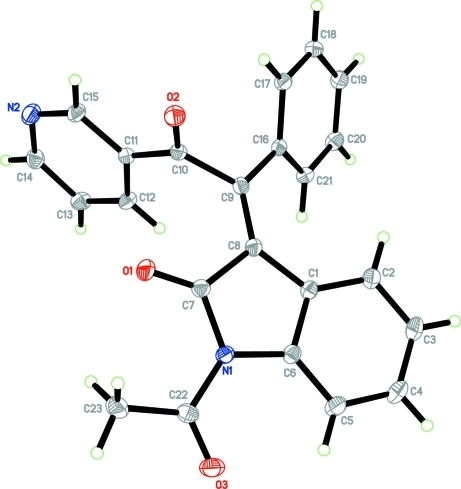
The molecular structure of the title compound, showing the atomic numbering scheme. Displacement ellipsoids for non-hydrogen atoms are drawn at the 50 % probability level.

**Fig. 2.**
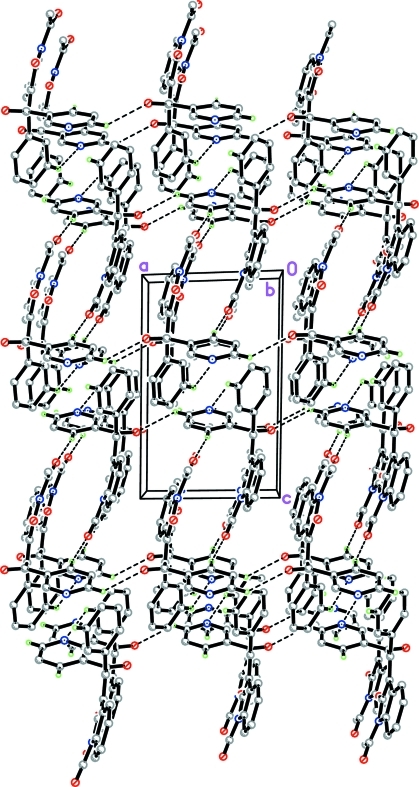
The crystal structure of the title compound, viewed along the b axis, showing a three-dimensional framework. H atoms not involved in intermolecular interactions (dashed lines) have been omitted for clarity.

## supplementary crystallographic information

### Comment

Indole derivatives have a variety of biological activities and are synthetic precursors for many naturally occuring alkaloids (Aanandhi *et al.*, 2008; Muthukumar *et al.*, 2008; Lawrence *et al.*, 2008). The C3 carbonyl group is the common reactive group in isatin (1H-indole-2,3-dione) for further derivation in either thermal reactions or photoreactions (Zhang *et al.*, 2004*b*; 2009). Photoreactions of *N*-acetylisatin and alkenes or alkynes have provided a facile way to build various heterocyclic frameworks containing indole moieties (Wang *et al.*, 2005; Zhang *et al.*, 2004*a*; Xue *et al.*, 2000). Azaaryl substituted acetylenes have been used to react with carbonyl groups which may lead to formation of various heterocyclic polycycles (Yu *et al.*, 2010). In view of the importance of the title compound, (I), as a typical quinone methide product in photoreaction between carbonyl and acetylenes, this paper reports its crystal structure.

The title molecule exists in a *cis* configuration with respect to the acyclic C8═C9 bond [C8═C9 = 1.3510 (14) Å]. The indoline ring system (C1-C8/N1) is essentially planar, with a maximum deviation of 0.080 (1) Å at atom C7. The phenyl ring (C16-C21) and pyridine ring (C11-C15/N2) are almost perpendicular to each other, as indicated by the interplanar angle of 88.93 (5)° between them. These two rings form dihedral angles of 72.97 (4) and 45.05 (4)° with the indoline ring system, respectively. The bond lengths and angles are consistent to those observed in closely related indole structures (Fun *et al.*, 2010**a*,b*; Usman *et al.*, 2001,2002).

In the crystal packing, intermolecular C12—H12A···O3, C14—H14A···O2 and C20—H20A···N2 hydrogen bonds (Table 1) interconnect adjacent molecules into a three-dimensional framework (Fig. 2). Weak intermolecular π-π interactions involving the pyrrolidine (C1/C6-C8/N1, centroid Cg1) and phenyl (C1-C6, centroid Cg2) rings [Cg1···Cg2^*^ = 3.6812 (11) Å; symmetry code: (*) 2-x, 2-y, -z] further stabilize the crystal packing.

### Experimental

The title compound was obtained in the reaction between *N*-acetylisatin and 3-pyridinyl phenyl acetylene under photo-irradiation with light of wavelength > 400 nm. The compound was purified by flash column chromatography with ethyl acetate-petroleum ether (1:5) as eluents. X-ray quality single crystals of (I) were obtained by slow evaporation of a acetone-petroleum ether (1:2) solution (m.p. 568–571 K).

### Refinement

H atoms were placed in their calculated positions, with C—H = 0.93 or 0.96 Å, and refined using a riding model, with *U*_iso_(H) = 1.2 or 1.5*U*_eq_(C). The rotating group model was applied to the methyl group.

### Crystal data

**Table:**

C_23_H_16_N_2_O_3_	*Z* = 2
*M**_r_* = 368.38	*F*(000) = 384
Triclinic, *P*1	*D*_x_ = 1.410 Mg m^−^^3^
Hall symbol: -P 1	Mo *K*α radiation, λ = 0.71073 Å
*a* = 7.9259 (19) Å	Cell parameters from 7658 reflections
*b* = 9.086 (2) Å	θ = 2.3–32.1°
*c* = 12.431 (3) Å	µ = 0.10 mm^−^^1^
α = 84.804 (7)°	*T* = 100 K
β = 87.064 (7)°	Block, yellow
γ = 76.843 (6)°	0.23 × 0.19 × 0.16 mm
*V* = 867.7 (4) Å^3^	

### Data collection

**Table:**

Bruker APEXII DUO CCD area-detector diffractometer	5017 independent reflections
Radiation source: fine-focus sealed tube	4237 reflections with *I* > 2σ(*I*)
graphite	*R*_int_ = 0.028
φ and ω scans	θ_max_ = 30.0°, θ_min_ = 1.7°
Absorption correction: multi-scan (*SADABS*; Bruker, 2009)	*h* = −11→11
*T*_min_ = 0.978, *T*_max_ = 0.985	*k* = −12→12
19600 measured reflections	*l* = −17→17

### Refinement

**Table:**

Refinement on *F*^2^	Primary atom site location: structure-invariant direct methods
Least-squares matrix: full	Secondary atom site location: difference Fourier map
*R*[*F*^2^ > 2σ(*F*^2^)] = 0.040	Hydrogen site location: inferred from neighbouring sites
*wR*(*F*^2^) = 0.116	H-atom parameters constrained
*S* = 1.04	*w* = 1/[σ^2^(*F*_o_^2^) + (0.0637*P*)^2^ + 0.2808*P*] where *P* = (*F*_o_^2^ + 2*F*_c_^2^)/3
5017 reflections	(Δ/σ)_max_ < 0.001
254 parameters	Δρ_max_ = 0.44 e Å^−^^3^
0 restraints	Δρ_min_ = −0.20 e Å^−^^3^

### Special details

**Table:**

Experimental. The crystal was placed in the cold stream of an Oxford Cryosystems Cobra open-flow nitrogen cryostat (Cosier & Glazer, 1986) operating at 100.0 (1)K.
Geometry. All esds (except the esd in the dihedral angle between two l.s. planes) are estimated using the full covariance matrix. The cell esds are taken into account individually in the estimation of esds in distances, angles and torsion angles; correlations between esds in cell parameters are only used when they are defined by crystal symmetry. An approximate (isotropic) treatment of cell esds is used for estimating esds involving l.s. planes.
Refinement. Refinement of F^2^ against ALL reflections. The weighted R-factor wR and goodness of fit S are based on F^2^, conventional R-factors R are based on F, with F set to zero for negative F^2^. The threshold expression of F^2^ > 2sigma(F^2^) is used only for calculating R-factors(gt) etc. and is not relevant to the choice of reflections for refinement. R-factors based on F^2^ are statistically about twice as large as those based on F, and R- factors based on ALL data will be even larger.

### Fractional atomic coordinates and isotropic or equivalent isotropic displacement parameters (Å^2^)

**Table:**

	*x*	*y*	*z*	*U*_iso_*/*U*_eq_	
O1	0.74656 (11)	0.64928 (9)	0.08150 (6)	0.01988 (17)	
O2	0.94194 (10)	0.51863 (9)	0.29214 (7)	0.01842 (17)	
O3	0.61891 (12)	0.98853 (10)	−0.16758 (7)	0.02319 (19)	
N1	0.72823 (12)	0.89175 (10)	−0.00538 (7)	0.01472 (18)	
N2	0.48355 (13)	0.38455 (11)	0.38814 (8)	0.0208 (2)	
C1	0.80969 (13)	1.00838 (12)	0.13505 (8)	0.01429 (19)	
C2	0.86200 (14)	1.12405 (12)	0.18121 (9)	0.0175 (2)	
H2A	0.8958	1.1110	0.2526	0.021*	
C3	0.86325 (15)	1.25906 (13)	0.11949 (10)	0.0200 (2)	
H3A	0.8961	1.3375	0.1501	0.024*	
C4	0.81564 (16)	1.27772 (13)	0.01211 (10)	0.0220 (2)	
H4A	0.8160	1.3692	−0.0279	0.026*	
C5	0.76744 (15)	1.16211 (13)	−0.03661 (9)	0.0199 (2)	
H5A	0.7379	1.1742	−0.1088	0.024*	
C6	0.76495 (13)	1.02835 (12)	0.02614 (8)	0.0149 (2)	
C7	0.75826 (13)	0.77964 (12)	0.08245 (8)	0.01466 (19)	
C8	0.79925 (13)	0.85485 (11)	0.17647 (8)	0.01383 (19)	
C9	0.80773 (13)	0.78123 (11)	0.27615 (8)	0.01317 (19)	
C10	0.80593 (13)	0.61403 (11)	0.29125 (8)	0.01361 (19)	
C11	0.63598 (13)	0.57473 (11)	0.31789 (8)	0.01344 (19)	
C12	0.47958 (14)	0.67902 (12)	0.29924 (9)	0.0165 (2)	
H12A	0.4784	0.7776	0.2711	0.020*	
C13	0.32642 (14)	0.63324 (13)	0.32333 (9)	0.0187 (2)	
H13A	0.2201	0.6996	0.3104	0.022*	
C14	0.33486 (14)	0.48613 (13)	0.36719 (9)	0.0192 (2)	
H14A	0.2312	0.4559	0.3831	0.023*	
C15	0.63067 (14)	0.42976 (12)	0.36275 (9)	0.0170 (2)	
H15A	0.7352	0.3606	0.3757	0.020*	
C16	0.80906 (13)	0.84819 (11)	0.38002 (8)	0.01300 (19)	
C17	0.90394 (13)	0.76491 (12)	0.46632 (8)	0.0148 (2)	
H17A	0.9728	0.6691	0.4568	0.018*	
C18	0.89565 (14)	0.82461 (12)	0.56573 (9)	0.0164 (2)	
H18A	0.9591	0.7688	0.6226	0.020*	
C19	0.79292 (14)	0.96743 (12)	0.58082 (9)	0.0173 (2)	
H19A	0.7894	1.0079	0.6472	0.021*	
C20	0.69532 (14)	1.04977 (12)	0.49644 (9)	0.0169 (2)	
H20A	0.6253	1.1448	0.5068	0.020*	
C21	0.70218 (13)	0.99052 (12)	0.39705 (9)	0.0150 (2)	
H21A	0.6355	1.0455	0.3412	0.018*	
C22	0.66296 (14)	0.87655 (13)	−0.10556 (9)	0.0168 (2)	
C23	0.65025 (17)	0.72265 (14)	−0.13229 (10)	0.0238 (2)	
H23D	0.6085	0.7288	−0.2041	0.036*	
H23A	0.5715	0.6849	−0.0817	0.036*	
H23B	0.7626	0.6552	−0.1283	0.036*	

### Atomic displacement parameters (Å^2^)

**Table:**

	*U*^11^	*U*^22^	*U*^33^	*U*^12^	*U*^13^	*U*^23^
O1	0.0294 (4)	0.0142 (4)	0.0177 (4)	−0.0079 (3)	−0.0024 (3)	−0.0016 (3)
O2	0.0157 (3)	0.0153 (4)	0.0234 (4)	−0.0011 (3)	−0.0024 (3)	−0.0019 (3)
O3	0.0304 (4)	0.0212 (4)	0.0168 (4)	−0.0038 (3)	−0.0049 (3)	0.0021 (3)
N1	0.0193 (4)	0.0125 (4)	0.0125 (4)	−0.0037 (3)	−0.0008 (3)	−0.0007 (3)
N2	0.0205 (5)	0.0161 (4)	0.0260 (5)	−0.0057 (4)	−0.0015 (4)	0.0014 (4)
C1	0.0149 (4)	0.0134 (4)	0.0147 (5)	−0.0037 (4)	0.0014 (3)	−0.0010 (3)
C2	0.0196 (5)	0.0168 (5)	0.0175 (5)	−0.0067 (4)	0.0016 (4)	−0.0029 (4)
C3	0.0230 (5)	0.0148 (5)	0.0239 (6)	−0.0076 (4)	0.0035 (4)	−0.0038 (4)
C4	0.0262 (6)	0.0145 (5)	0.0250 (6)	−0.0062 (4)	0.0030 (4)	0.0016 (4)
C5	0.0248 (5)	0.0167 (5)	0.0176 (5)	−0.0048 (4)	0.0002 (4)	0.0017 (4)
C6	0.0166 (4)	0.0132 (4)	0.0147 (5)	−0.0035 (4)	0.0015 (3)	−0.0017 (4)
C7	0.0167 (4)	0.0141 (4)	0.0130 (4)	−0.0033 (4)	−0.0004 (3)	−0.0008 (4)
C8	0.0150 (4)	0.0129 (4)	0.0141 (5)	−0.0038 (3)	−0.0006 (3)	−0.0017 (3)
C9	0.0121 (4)	0.0126 (4)	0.0151 (5)	−0.0032 (3)	−0.0010 (3)	−0.0015 (3)
C10	0.0157 (4)	0.0128 (4)	0.0122 (4)	−0.0028 (4)	−0.0022 (3)	−0.0006 (3)
C11	0.0149 (4)	0.0126 (4)	0.0131 (4)	−0.0035 (4)	−0.0015 (3)	−0.0009 (3)
C12	0.0168 (5)	0.0135 (4)	0.0182 (5)	−0.0022 (4)	−0.0006 (4)	0.0017 (4)
C13	0.0142 (4)	0.0191 (5)	0.0213 (5)	−0.0015 (4)	−0.0010 (4)	0.0013 (4)
C14	0.0177 (5)	0.0207 (5)	0.0204 (5)	−0.0074 (4)	−0.0001 (4)	−0.0010 (4)
C15	0.0159 (5)	0.0129 (4)	0.0213 (5)	−0.0020 (4)	−0.0024 (4)	0.0007 (4)
C16	0.0136 (4)	0.0130 (4)	0.0130 (4)	−0.0048 (3)	−0.0002 (3)	−0.0004 (3)
C17	0.0154 (4)	0.0135 (4)	0.0154 (5)	−0.0031 (4)	−0.0007 (4)	−0.0003 (4)
C18	0.0181 (5)	0.0175 (5)	0.0138 (5)	−0.0047 (4)	−0.0028 (4)	0.0009 (4)
C19	0.0213 (5)	0.0176 (5)	0.0139 (5)	−0.0059 (4)	0.0013 (4)	−0.0028 (4)
C20	0.0194 (5)	0.0138 (4)	0.0169 (5)	−0.0026 (4)	0.0025 (4)	−0.0024 (4)
C21	0.0159 (4)	0.0139 (4)	0.0145 (5)	−0.0030 (4)	−0.0004 (3)	0.0006 (4)
C22	0.0169 (5)	0.0194 (5)	0.0136 (5)	−0.0023 (4)	0.0002 (4)	−0.0030 (4)
C23	0.0328 (6)	0.0205 (5)	0.0189 (5)	−0.0051 (5)	−0.0066 (4)	−0.0047 (4)

### Geometric parameters (Å, °)

**Table:**

O1—C7	1.2100 (13)	C11—C15	1.3919 (15)
O2—C10	1.2196 (13)	C11—C12	1.3940 (14)
O3—C22	1.2146 (14)	C12—C13	1.3807 (15)
N1—C22	1.4027 (14)	C12—H12A	0.93
N1—C7	1.4146 (13)	C13—C14	1.3854 (16)
N1—C6	1.4293 (14)	C13—H13A	0.93
N2—C15	1.3360 (15)	C14—H14A	0.93
N2—C14	1.3421 (15)	C15—H15A	0.93
C1—C2	1.3920 (15)	C16—C17	1.4020 (14)
C1—C6	1.4025 (15)	C16—C21	1.4026 (14)
C1—C8	1.4614 (14)	C17—C18	1.3866 (15)
C2—C3	1.3884 (16)	C17—H17A	0.93
C2—H2A	0.93	C18—C19	1.3890 (15)
C3—C4	1.3909 (17)	C18—H18A	0.93
C3—H3A	0.93	C19—C20	1.3914 (15)
C4—C5	1.3920 (17)	C19—H19A	0.93
C4—H4A	0.93	C20—C21	1.3855 (15)
C5—C6	1.3868 (15)	C20—H20A	0.93
C5—H5A	0.93	C21—H21A	0.93
C7—C8	1.4911 (14)	C22—C23	1.4925 (16)
C8—C9	1.3510 (14)	C23—H23D	0.96
C9—C16	1.4779 (14)	C23—H23A	0.96
C9—C10	1.5169 (15)	C23—H23B	0.96
C10—C11	1.4844 (15)		
			
C22—N1—C7	126.23 (9)	C13—C12—H12A	120.6
C22—N1—C6	124.62 (9)	C11—C12—H12A	120.6
C7—N1—C6	109.05 (8)	C12—C13—C14	118.45 (10)
C15—N2—C14	116.88 (10)	C12—C13—H13A	120.8
C2—C1—C6	119.44 (10)	C14—C13—H13A	120.8
C2—C1—C8	132.38 (10)	N2—C14—C13	123.95 (10)
C6—C1—C8	108.08 (9)	N2—C14—H14A	118.0
C3—C2—C1	119.30 (10)	C13—C14—H14A	118.0
C3—C2—H2A	120.3	N2—C15—C11	123.54 (10)
C1—C2—H2A	120.3	N2—C15—H15A	118.2
C2—C3—C4	120.41 (10)	C11—C15—H15A	118.2
C2—C3—H3A	119.8	C17—C16—C21	118.84 (10)
C4—C3—H3A	119.8	C17—C16—C9	120.64 (9)
C3—C4—C5	121.29 (10)	C21—C16—C9	120.24 (9)
C3—C4—H4A	119.4	C18—C17—C16	120.34 (10)
C5—C4—H4A	119.4	C18—C17—H17A	119.8
C6—C5—C4	117.78 (11)	C16—C17—H17A	119.8
C6—C5—H5A	121.1	C17—C18—C19	120.29 (10)
C4—C5—H5A	121.1	C17—C18—H18A	119.9
C5—C6—C1	121.74 (10)	C19—C18—H18A	119.9
C5—C6—N1	128.68 (10)	C18—C19—C20	119.87 (10)
C1—C6—N1	109.51 (9)	C18—C19—H19A	120.1
O1—C7—N1	126.06 (10)	C20—C19—H19A	120.1
O1—C7—C8	127.05 (10)	C21—C20—C19	120.17 (10)
N1—C7—C8	106.84 (9)	C21—C20—H20A	119.9
C9—C8—C1	133.76 (10)	C19—C20—H20A	119.9
C9—C8—C7	119.91 (10)	C20—C21—C16	120.45 (10)
C1—C8—C7	106.16 (9)	C20—C21—H21A	119.8
C8—C9—C16	126.82 (10)	C16—C21—H21A	119.8
C8—C9—C10	120.64 (9)	O3—C22—N1	119.12 (10)
C16—C9—C10	112.44 (9)	O3—C22—C23	122.22 (10)
O2—C10—C11	122.38 (10)	N1—C22—C23	118.65 (10)
O2—C10—C9	120.09 (9)	C22—C23—H23D	109.5
C11—C10—C9	117.10 (9)	C22—C23—H23A	109.5
C15—C11—C12	118.37 (10)	H23D—C23—H23A	109.5
C15—C11—C10	119.66 (9)	C22—C23—H23B	109.5
C12—C11—C10	121.97 (9)	H23D—C23—H23B	109.5
C13—C12—C11	118.78 (10)	H23A—C23—H23B	109.5
			
C6—C1—C2—C3	2.16 (16)	C16—C9—C10—O2	−91.54 (12)
C8—C1—C2—C3	177.86 (11)	C8—C9—C10—C11	−95.54 (12)
C1—C2—C3—C4	−1.10 (17)	C16—C9—C10—C11	81.14 (11)
C2—C3—C4—C5	−0.66 (18)	O2—C10—C11—C15	10.99 (16)
C3—C4—C5—C6	1.30 (17)	C9—C10—C11—C15	−161.52 (10)
C4—C5—C6—C1	−0.20 (17)	O2—C10—C11—C12	−168.92 (10)
C4—C5—C6—N1	−176.92 (10)	C9—C10—C11—C12	18.58 (15)
C2—C1—C6—C5	−1.54 (16)	C15—C11—C12—C13	−1.65 (16)
C8—C1—C6—C5	−178.19 (10)	C10—C11—C12—C13	178.26 (10)
C2—C1—C6—N1	175.75 (9)	C11—C12—C13—C14	1.31 (16)
C8—C1—C6—N1	−0.91 (12)	C15—N2—C14—C13	−1.21 (18)
C22—N1—C6—C5	−9.52 (17)	C12—C13—C14—N2	0.14 (18)
C7—N1—C6—C5	173.99 (11)	C14—N2—C15—C11	0.83 (17)
C22—N1—C6—C1	173.44 (9)	C12—C11—C15—N2	0.58 (17)
C7—N1—C6—C1	−3.05 (12)	C10—C11—C15—N2	−179.33 (10)
C22—N1—C7—O1	6.86 (17)	C8—C9—C16—C17	−146.10 (11)
C6—N1—C7—O1	−176.72 (10)	C10—C9—C16—C17	37.46 (13)
C22—N1—C7—C8	−170.80 (9)	C8—C9—C16—C21	40.03 (15)
C6—N1—C7—C8	5.61 (11)	C10—C9—C16—C21	−136.40 (10)
C2—C1—C8—C9	13.1 (2)	C21—C16—C17—C18	−1.83 (15)
C6—C1—C8—C9	−170.79 (11)	C9—C16—C17—C18	−175.78 (9)
C2—C1—C8—C7	−171.80 (11)	C16—C17—C18—C19	0.13 (16)
C6—C1—C8—C7	4.26 (11)	C17—C18—C19—C20	1.24 (16)
O1—C7—C8—C9	−7.79 (17)	C18—C19—C20—C21	−0.87 (17)
N1—C7—C8—C9	169.84 (9)	C19—C20—C21—C16	−0.86 (16)
O1—C7—C8—C1	176.33 (11)	C17—C16—C21—C20	2.19 (15)
N1—C7—C8—C1	−6.04 (11)	C9—C16—C21—C20	176.17 (10)
C1—C8—C9—C16	8.63 (19)	C7—N1—C22—O3	168.91 (10)
C7—C8—C9—C16	−165.88 (9)	C6—N1—C22—O3	−6.97 (16)
C1—C8—C9—C10	−175.20 (10)	C7—N1—C22—C23	−11.15 (16)
C7—C8—C9—C10	10.28 (15)	C6—N1—C22—C23	172.96 (10)
C8—C9—C10—O2	91.78 (13)		

### Hydrogen-bond geometry (Å, °)

**Table:**

*D*—H···*A*	*D*—H	H···*A*	*D*···*A*	*D*—H···*A*
C12—H12A···O3^i^	0.93	2.37	3.2518 (16)	158
C14—H14A···O2^ii^	0.93	2.53	3.2381 (16)	133
C20—H20A···N2^iii^	0.93	2.58	3.3238 (17)	137

Symmetry codes: (i) −*x*+1, −*y*+2, −*z*; (ii) *x*−1, *y*, *z*; (iii) *x*, *y*+1, *z*.
